# Synthesis of cesium lead halide perovskite/zinc oxide (CsPbX_3_/ZnO, X= Br, I) as heterostructure photocatalyst with improved activity for heavy metal degradation

**DOI:** 10.3389/fchem.2022.1020484

**Published:** 2022-10-05

**Authors:** Sehrish Gull, Saima Batool, Guijun Li, Muhammad Idrees

**Affiliations:** ^1^ Key Laboratory of Optoelectronics Devices and Systems of Ministry of Education, College of Physics and Optoelectronic Engineering, Shenzhen University, Shenzhen, China; ^2^ Institute for Advanced Study, Shenzhen University, Shenzhen, China; ^3^ Institute of Microscale Optoelectronics, Shenzhen University, Shenzhen, China; ^4^ Additive Manufacturing Institute, College of Mechatronics and Control Engineering, Shenzhen University, Shenzhen, China

**Keywords:** cesium lead halide/zinc oxide, heterostructure, hot injection strategy, photocatalyst, degradation

## Abstract

Inorganic perovskites have been recognized as highly potent materials for the display and medical industries due to their outstanding features. However, there haven’t been many reports on their implications as a photocatalyst for the removal of heavy metals. Photocatalysis has been regarded as a significant approach for the removal of pollutants because of its great sustainability, improved efficiency, and reduced energy consumption. Here, we applied inorganic cesium lead halides (Br and I) with zinc oxide heterostructure as a photocatalyst for the first time. The heterostructure has been synthesized by the traditional hot injection strategy and its photocatalytic activity was systematically investigated. Interestingly, the CsPbX_3_/ZnO heterostructure as a photocatalyst has a homogeneous geometry and possesses an excellent degradation efficiency of over 50% under xenon UV-Visible light. The CsPbX_3_/ZnO catalyst carries superior oxidation/reduction properties and ionic conductivity due to the synergistic photogenerated charge carrier and interaction between CsPbX_3_ and ZnO. The recycling experiment showed the good stability of the catalysts. These findings suggest that inorganic lead halide heterostructure has the potential to be used for heavy metal degradation and water pollution removal catalysts.

## 1 Introduction

Lead tri-halide perovskites have been considered as a fascinating class of materials for next generation applications due to their remarkable characteristics, including enhanced optical features, high extinction constant, tunable bandgap, versatile surface chemistry, long-range electron-hole diffusion, and high carrier mobility (Akkerman et al., 2015; Gull et al., 2020). This class of materials possesses the general formula of ABX_3_ where A is a cation (organic/inorganic), B is a divalent metal (Pb^2+^, Sn^2+^, Ge^2+^), and X is an anion (Cl^1-^, Br^1-^, I^1-^ or mixture) (Kulkarni et al., 2019; Yang et al., 2019). In recent years, semiconductor materials have been widely used as photocatalyst in the environmental and energy sectors due to their novel physiochemical features and cost-effectiveness (Batool et al., 2020; Shen et al., 2021b) Commonly used metal-based semiconducting photocatalysts include TiO_2_ (Fujishima et al., 2000) (Zhang et al., 2021b), Fe_2_O_3_ (Hitam and Jalil, 2020), CdS (Cheng et al., 2018), MoS_2_ (Shen et al., 2020), ZnS (Lee and Wu, 2017), and ZnO (Johar et al., 2015).

Zinc Oxide (ZnO) has a bandgap of 3.37 eV and can treat heavy metals due to its high photocatalytic efficiency and excitation binding energy-producing electron-hole pair (ehp) under UV or visible irradiation (Huang et al., 2015; Le et al., 2019). The electron and hole combine with the adsorbed oxygen (O^.^) on the photocatalyst surface and water (H_2_O) to generate O_2_ and hydroxyl (OH), which help in the oxidation of organic products into end products (CO_2_ and H_2_O) (Senapati et al., 2012). Photocatalysis has been regarded as a significant approach for pollutant removal due to its high sustainability, improved efficiency, and low energy consumption (Shen et al., 2021a; Li et al., 2022b) The investigation of photocatalysts with remarkable features such as extraordinary sunlight absorption, significant generation, and separation of charge carriers with enhanced redox potential is quite useful for achieving efficient photocatalytic removal of pollutant (Idrees et al., 2016; Li et al., 2022c). Photocatalysts, a green technology that uses solar energy, have a significant impact on environmental restoration. Therefore, more time is required to investigate potential photocatalysts (Li et al., 2022a; Cai et al., 2022; Li et al., 2022d). In this aspect, lead tri-halide with unique properties could also be used in the photochemical conversion, if the issues of stability, inefficient photocatalytic activity, and rigorous ehp recombination rate could be optimized (Zhao et al., 2020). There have recently been a few reports on the use of inorganic lead tri-halide perovskites, their derivatives, and composites as photocatalysts. Among the cesium-based lead halides, it was observed that pristine bromide-based compounds with a wide-bandgap are difficult to photocatalyze; however, after different treatments such as making a heterostructure or changing the typical ligands are an optimal choice (Xu et al., 2017). Moreover, photoreduction of CO_2_ and hydrogen evolution using a mixture of Br/Cl and Br/I has been reported (Guan et al., 2019). In comparison to these inorganic halides, cesium lead iodide (CsPbI_3_) had a narrow bandgap of 1.73 eV, high emission intensity, and existed in two major phases known as alpha phase (α-CsPbI_3_) and delta phase (δ-CsPbI_3_) (Lai et al., 2017). Lin et al., recently reported the use of CsPbI_3_ as a photocatalyst by making heterostructure with tungsten disulfide (WS_2_), where they used γ-CsPbI_3_ nanocrystals fabricated with several layered tungsten disulfides for the complete degradation of methylene blue (MB) into less toxic inorganic products with high photocatalytic degradation efficiency (Zhang et al., 2019). The coupling of inorganic perovskites with other compounds is considered as useful strategy for addressing the issues of instability, photocatalytic activity deficiency, and controlled ehp recombination. Ma et al. reported on the photocatalytic activity of graphitic carbon nitride (g-C_3_N_4_) combined with CsPbI_3_ for photocatalytic degradation of the organic dye rhodamine B, and they also reported the use of Pt as a co-catalyst for hydrogen generation by water splitting (Liu and Ma, 2021). Up to now, there are no reports about the photocatalytic degradation of inorganic pollutants from water streams by the CsPbX_3_/ZnO heterostructure.

Here, for the first time, we used CsPbX_3_/ZnO heterostructure with ZnO as a photocatalyst to study the photocatalytic activity of CsPbX_3_. (Zhang et al., 2021a). Although Xu et al., recently reported a CsPbX_3_/ZnO heterostructure for light-emitting diodes. Wang et al., also used ZnO as a basis for transporting electrons to solar cells (Deng et al., 2020). To date, however, no reports have been found regarding the use of ZnO and CsPbX_3_ for photocatalytic applications. This study is aimed at modifying CsPbI_3_, and CsPbBr_3_ using ZnO with the desired properties for the efficient photo degradation of heavy metals. We investigated the photocatalytic response in CsPbI_3_, CsPbBr_3_ forming heterostructures with ZnO and the results proved that δ-CsPbI_3_/ZnO heterostructure is quite beneficial in the photo degradation of heavy metals under visible light with an efficiency of more than 50%, owing to its hexagonal structure.

## 2 Materials and method

### 2.1 Materials

Cesium carbonate (Cs_2_CO_3_, 99.99%) was bought from Macklin. Lead iodide (PbI_2_, 99.99%), Lead bromide (PbBr_2_, 99.99%), Octadecene (ODE, technical grade 90%), Oleic acid (OA, technical grade 90%), oleylamine (OAm, technical grade 70%), Zinc Stearate (ZnSt_2_, 98%), Toluene (98%) and Hexane (99.9%) were purchased from Sigma Aldrich and used as it is received without any further modification.

### 2.2 Photocatalysts synthesis

#### 2.2.1 Formation of cesium and lead halide precursors by hot injection method

**FIGURE 1:**
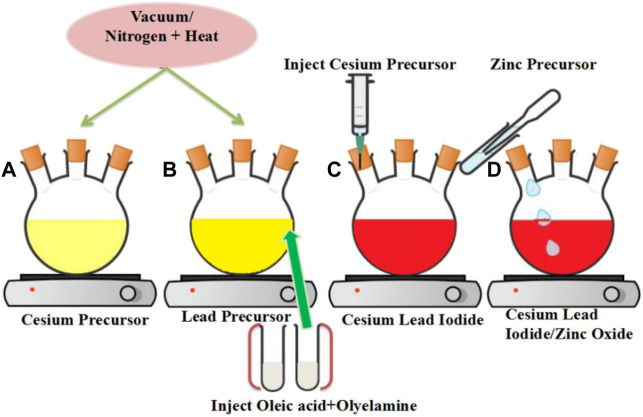
Schematic representation for the synthesis of the CsPbI_3_/ZnO heterostructure **(A)** Cesium precursor **(B)** Lead precursor **(C)** formation of Cesium Lead Iodide **(D)** formation of Cesium Lead Iodide/Zinc Oxide heterostructure.

Protesescu’s hot injection strategy was used for the synthesis of CsPbX_3_/ZnO heterostructure, with minor modifications (Protesescu et al., 2015). The stepwise schematic presentation is shown in Figure 1. Firstly, 0.6 g of cesium carbonate (Cs_2_CO_3_), 2 ml OA, and 20 ml ODE were added to a 50 ml 3-neck round bottom flask and stirred continuously under vacuum for 30 min at 125°C, absolving the flask with nitrogen (N_2_) for 10 min, it was placed back under vacuum. An alternative implication of vacuum and N_2_ to completely remove moisture and oxygen has been applied as shown in Figure 1A. The PbI_2_/Br_2_ precursors were then synthesized by degassing 0.8 g of PbI_2_ and 0.6 g of PbBr_2_ in 20 ml ODE for 1 h under constant stirring and heating at 125°C in a 50 ml flask. The flask was then filled with a 1:1 mixture of OA and OAm (4 ml each, pre-heated at ∼70°C) and vacuumed again for 15–30 min, until the salt of lead halides was completely dissolved and the solution was no longer releasing gas (15–30 min), as shown in Figure 1B.

### 2.3 Formation of cesium lead halide/zinc oxide heterostructure

For the formation of cesium lead halides, the temperature of the lead halide precursor was raised to 150°C, and 4 ml cesium precursor was injected swiftly under a nitrogen environment, as shown in Figure 1C. Later, for the photocatalytic activity investigation of as synthesized CsPbX_3_ with Zinc oxide, 0.4 mg ml^−1^ zinc stearate in ODE was injected into the above mixture as a zinc source for the formation of CsPbX_3_/ZnO heterostructure as shown in Figure 1D, aliquotes were taken at different times, and the reaction was quenched by dipping the flask into the ice bath. Finally, the solution was centrifuged for 3 min at 10,000 rpm, the supernatant was discarded, and precipitates were dispersed in toluene/hexane for further investigation.

### 2.4 Characterization techniques

X-ray diffraction patterns (XRD) spectra was collected X-ray diffractometer (Bruker D8 advance, Germany) with Cu K_α_ radiation (*k* = 1.54 nm) in the range of 2θ = 20°–70°. The valence properties of all existing elements in CsPbX_3_/ZnO NPs were determined by employing X-ray photoelectron (XPS: ESCALAB 250Xi-system) Ultraviolet-visible (UV-visible) spectra were investigated by a Lambda 950 spectrophotometer in the wavelength range of 300–800 nm. Fourier Transform Infrared (FTIR) has been carried out by Shimadzu-8400S infrared spectrometer. The morphology of the material was investigated by transmission electron microscope (JEOL JSM-7800F) and energy-dispersive spectra (EDS) was obtained by using an integrated Oxford INCA X-ACT equipped with SEM. On a multi-channel battery system (LANHE-CT2001A), the electrocatalytic activity of prototype coin cells was studied in a voltage range of 0.1–3.0 V at a constant current density.

### 2.5 Photocatalytic activity measuring experiment

The photocatalytic activity of CsPbX_3_/ZnO heterostrucutre as a photocatalyst was irradiated to a 300W xenon lamp in ambient conditions. For which ZnO (0.4 mgmL^−1^ ZnSt_2_ in ODE) was injected to CsPbX_3_ solution, under constant magnetic stirring and heating to achieve equilibrium. For the photocatalytic inquiry, two different temperatures have been used i.e., 100°C and 160°C. After injecting zinc precursor into the halide perovskites, samples were prepared using 15 µL (CsPbX_3_/ZnO) in 1 ml hexane at different time intervals starting from 2 min till 30min. Photocatalytic activity under UV-visible spectrophotometer in the wavelength range of 300–800 nm has been observed. To explore the photocatalytic activity of ZnO as a photocatalyst, the absorbance spectra of halide perovskites with and without zinc oxide were compared.

Degradation efficiency (%) has been calculated by using Eq. 1.

$$Degradation\;\left(\%\right)=\left[\left(C_{o}-C_{t}\right)/C_{o}\right]*100,$$ (1)

where C_o_ is degradation concentration without ZnO and C_t_ degradation concentration with ZnO at different intervals in the above relation (Huang et al., 2015; Sajid et al., 2020).

## 3 Results and discussions

### 3.1 Characterization of CsPbX_3_/ZnO heterostructure

**FIGURE 2:**
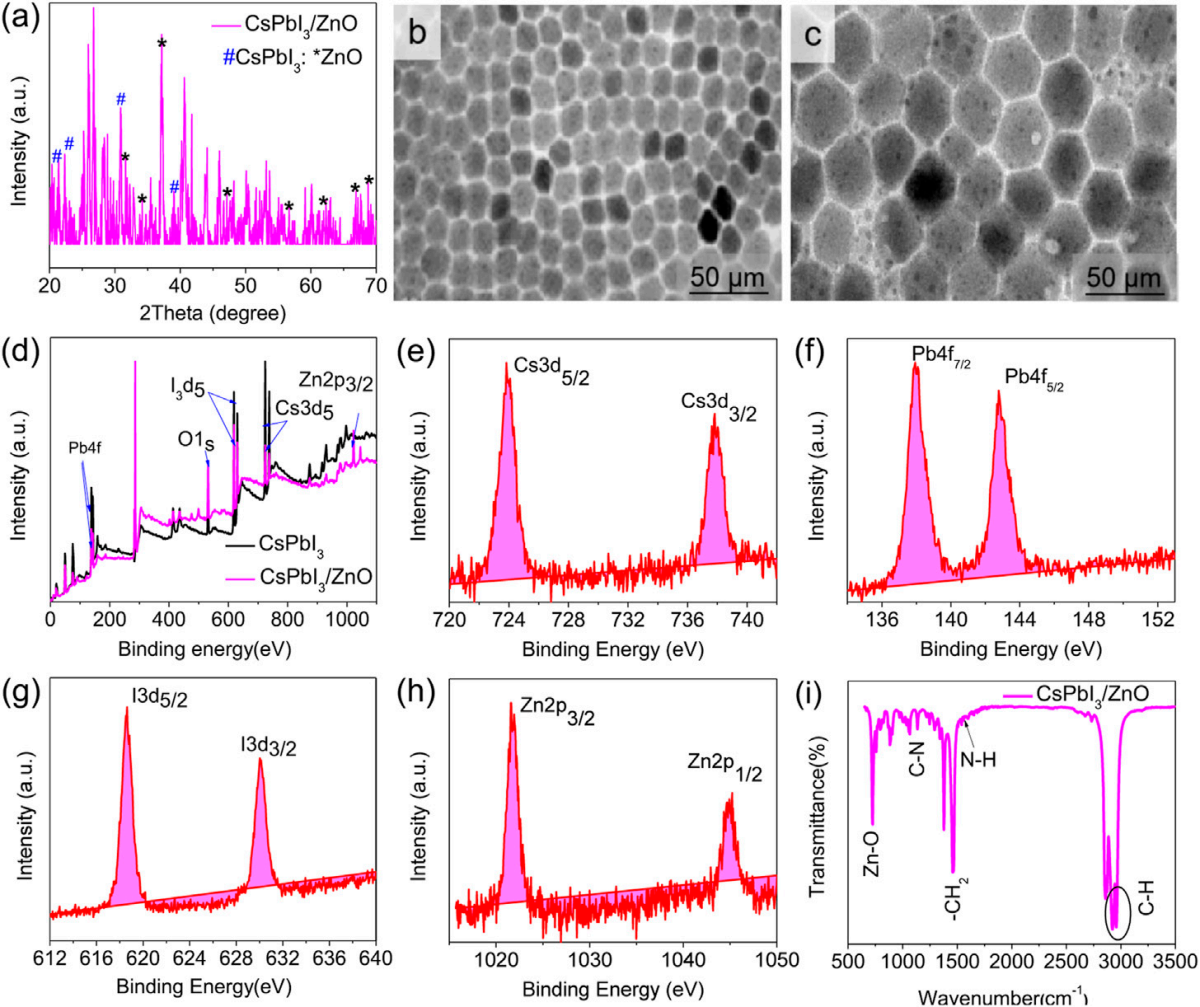
**(A)** X-ray diffraction (XRD) pattern of CsPbI_3_/ZnO heterostructure**, (B,C)** TEM images of CsPbI_3_ and CsPbI_3_/ZnO heterostructure. **(D)** The general XPS spectrum and **(E–H)** HR-XPS spectrum of CsPbI_3_/ZnO heterostructure. **(I)** FTIR spectra of CsPbI_3_/ZnO heterostructure.

Studies have shown that inorganic cesium lead halide perovskite is considered as a hot area for scientists and researchers due to its outstanding performance in different applications. Different reports highlight its usage as photocatalysts for CO_2_ reduction (Wang et al., 2019), hydrogen evolution (Zhao et al., 2021) and degradation of different pollutants from the environment and industry (Zhao et al., 2020; Li et al., 2023). To investigate the as-synthesized system of CsPbX_3_/ZnO heterostructure, different investigations have been carried out. Figure 2A is the XRD pattern of the CsPbI_3_/ZnO heterostructure. The XRD pattern confirmed the presence of both CsPbI_3_ and ZnO, which are highlighted with different symbols. All of the characteristic peaks correspond to JCPDS card number. 18–0376 and 36–1,451, which represent the delta and wurtzite phases, respectively. The diffraction peaks located at 21.6°, 22.7°,31.3°, and 39.3° indicate the presence of CsPbI_3_ (highlighted with #), whereas the peaks at 31.8°,34.4°, 36.2°, 47.5°, 56.5°, 62.8°, 66.3°, and 67.9° correspond to the ZnO (represented with *) (Zhang et al., 2007). Figures 2B,C show the morphology of CsPbI_3_ and CsPbI_3_/ZnO heterostructures having the hexagonal geometry of CsPbI_3_ with an average particle size of 28.3 nm that has been enlarged to 34.5 nm after the addition of photocatalyst ZnO, proving the inclusion of ZnO with the retention of the same hexagonal morphology. The dots on the surface of Figure 2C justifying the formation of the CsPbI_3_/ZnO heterostructure.

XPS analysis was conducted to investigate the chemical states of elements presented in the as-synthesized CsPbI_3_/ZnO heterostructure (Figure 2D). The general XPS survey spectrum proves the existence of Cs, Pb, I, Zn, and O, confirming the formation of CsPbI_3_/ZnO. Figures 2E–H presents the HR-XPS deconvoluted peaks of all existing elements.

The HR-XPS Cs spectrum deconvoluted into peaks at 723.86 and 737.83 eV, ascribed to the Cs3d of Cs3d_5/2_ and Cs3d_3/2_, respectively (Figure 2E). The peaks at 137.96 and 142.80 eV in the core level spectrum of Pb4f are attributed to Pb4f_7/2_ and Pb4f_5/2_, respectively (Figure 2F). Similarly, the core-level I3d spectrum position at 618.61 eV indicated the presence of I3d_5/2_ and 630 eV I3d_3/2_ (Figure 2G), while the peaks at 1,021.9 and 1,044.94 eV were attributed to the formation of Zn2p_3/2_ and Zn2p_1/2_ (Zn-O), respectively (Figure 2H) (Lu et al., 2018; Pirhashemi et al., 2019).

**FIGURE 3:**
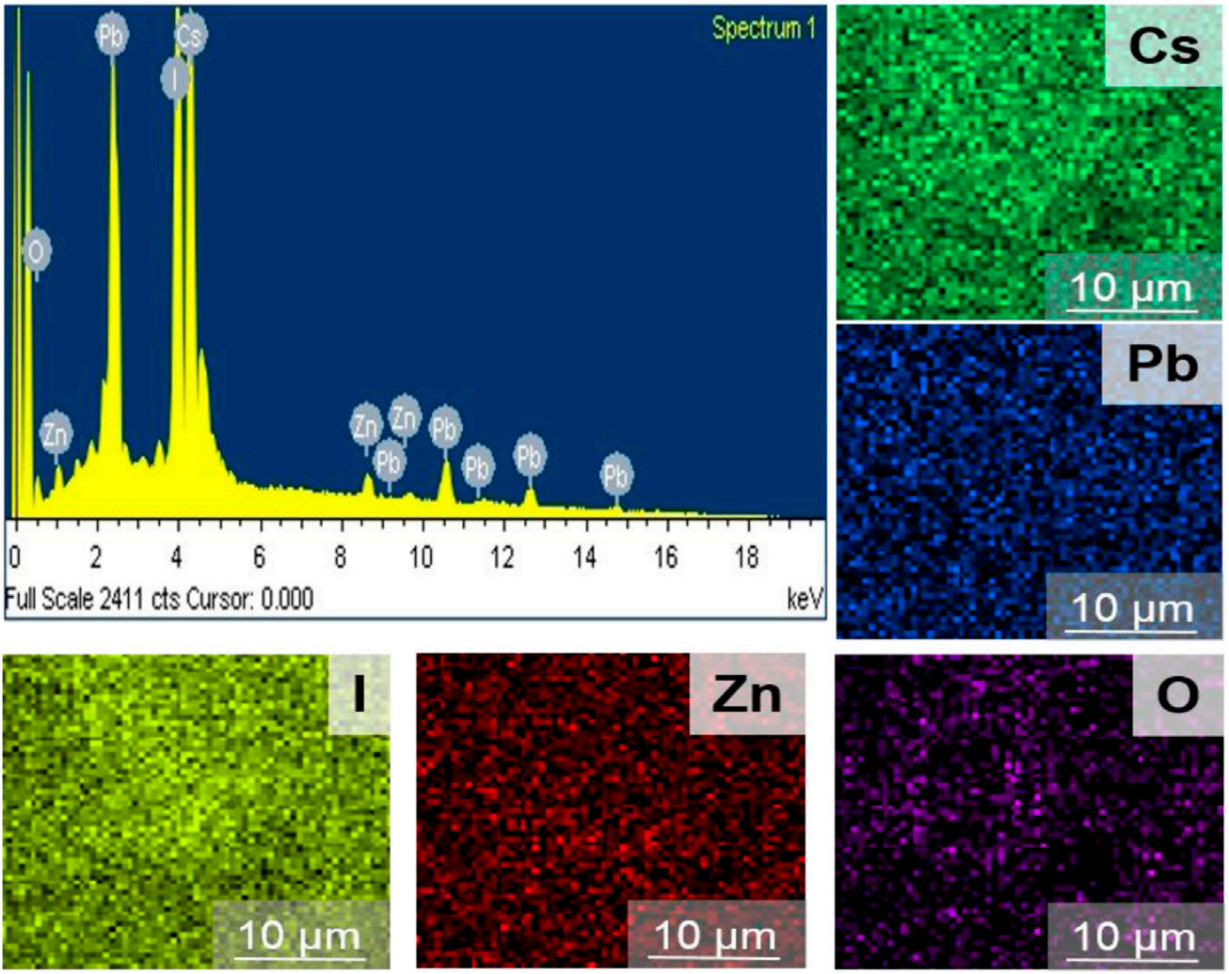
EDS spectra of CsPbI_3_/ZnO heterostructure and its corresponding elements’ EDS mapping.

The surface functionality of the materials was recorded by FTIR spectrum in the range of 650–3,500 cm^−1^. Figure 2I shows the FTIR spectra of the CsPbI_3_/ZnO heterostructure in which the peak at 716 cm^−1^ shows the bonding between Zn-O, whereas the rest of the peaks show the presence of different ligands used for the formation of heterostructure. Generally, the peak at 1,153 cm^−1^ represents C-N, 1,473 cm^−1^ shows -CH_2_, 1,641 cm^−1^ to N-H, and 2,913 and 2,955 cm^−1^ correspond to the presence of C-H attributed due to the aliphatic chains of octadecyl, oleylamine and oleic acid (An et al., 2018) (Nipane et al., 2013). EDS was used to investigate the elemental percentages in the synthesized compound, as shown in Figure 3.

The EDS spectrum confirms the existence and equal distribution of all the elements in the synthesized compound, and their atomic percentage is listed in Table 1.

**TABLE 1:** Elemental percentage of as-synthesized CsPbI_3_/ZnO heterostructure.

Elements	Atomic percentages (%)
Cs	16.78
Pb	14.40
I	55.02
Zn	6.27
O	7.53

### 3.2 Photocatalytic activity of CsPbX_3_/ZnO heterostructure

The basic photocatalytic mechanism starts with the generation of electrons and holes generated as a result of light irradiation with a wavelength greater than or equal to their bandgap; photo-induced electrons move from the valence band (VB) to the conduction band (CB), and corresponding holes move to the valence band. Hence, a redox reaction was carried out using these isolated electron-hole pairs (Ren et al., 2022). The as-synthesized product was optically characterized using UV-visible spectroscopy to analyze the behavior of inorganic halide perovskites with ZnO as a photocatalyst. The designed composite heterostructure is made up of interfaces made of various materials that are tightly bonded and have indistinguishable interface junctions (Zhao et al., 2018). The benefit of using zinc is that it has the capability to replace lead during the formation of heterostructure, indicating the potential to be used as a heavy metal degradation and water pollution removal catalyst (Kar et al., 2022).

**FIGURE 4:**
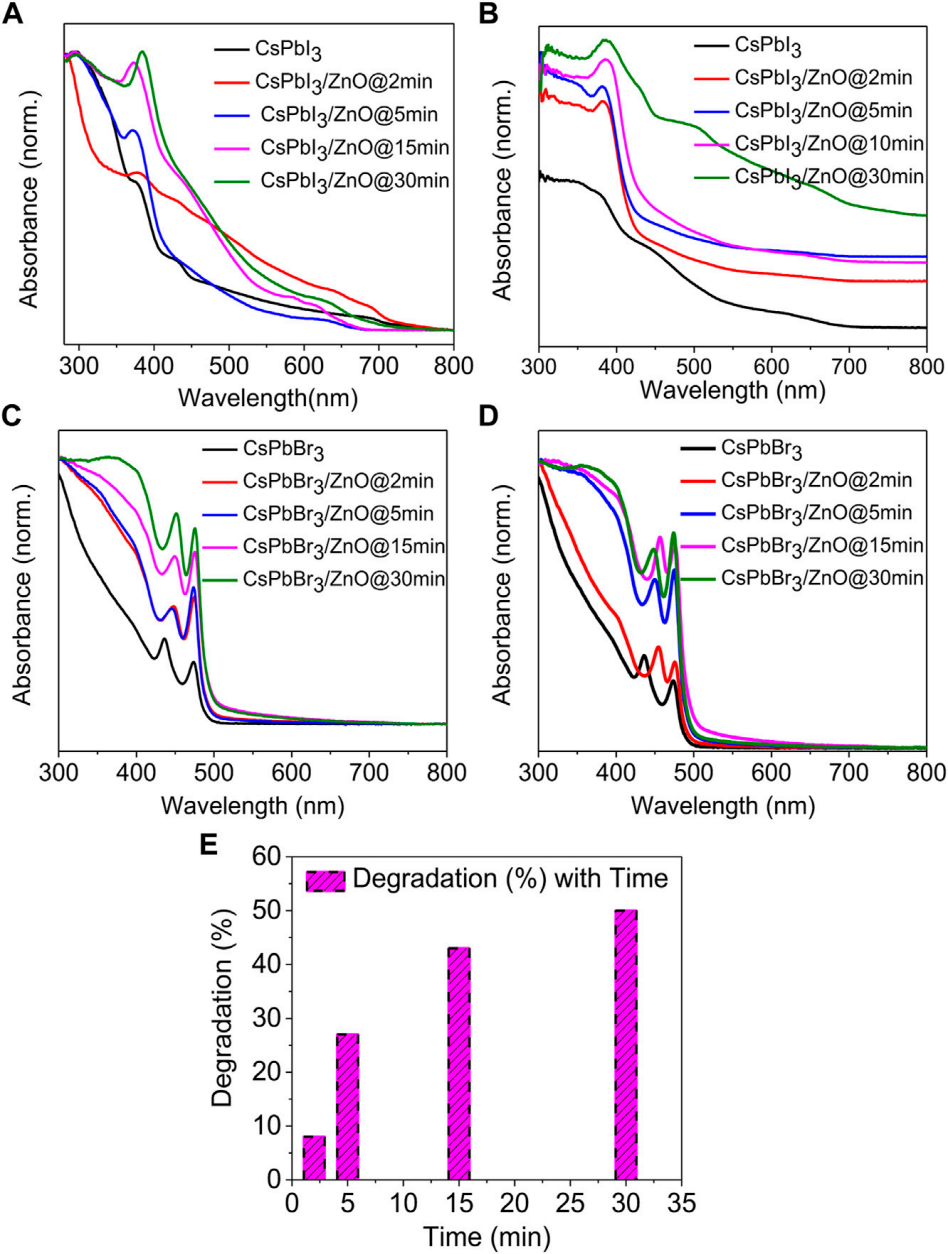
UV-visible absorbance spectra of CsPbI_3_ and CsPbI_3_/ZnO as a function of time at **(A)** 100°C and **(B)** 160°C. CsPbBr_3_ and CsPbBr_3_/ZnO at **(C)** 100°C and **(D)** 160°C. **(E)** Degradation efficiency with time for CsPbI_3_/ZnO heterostructure.

**FIGURE 5:**
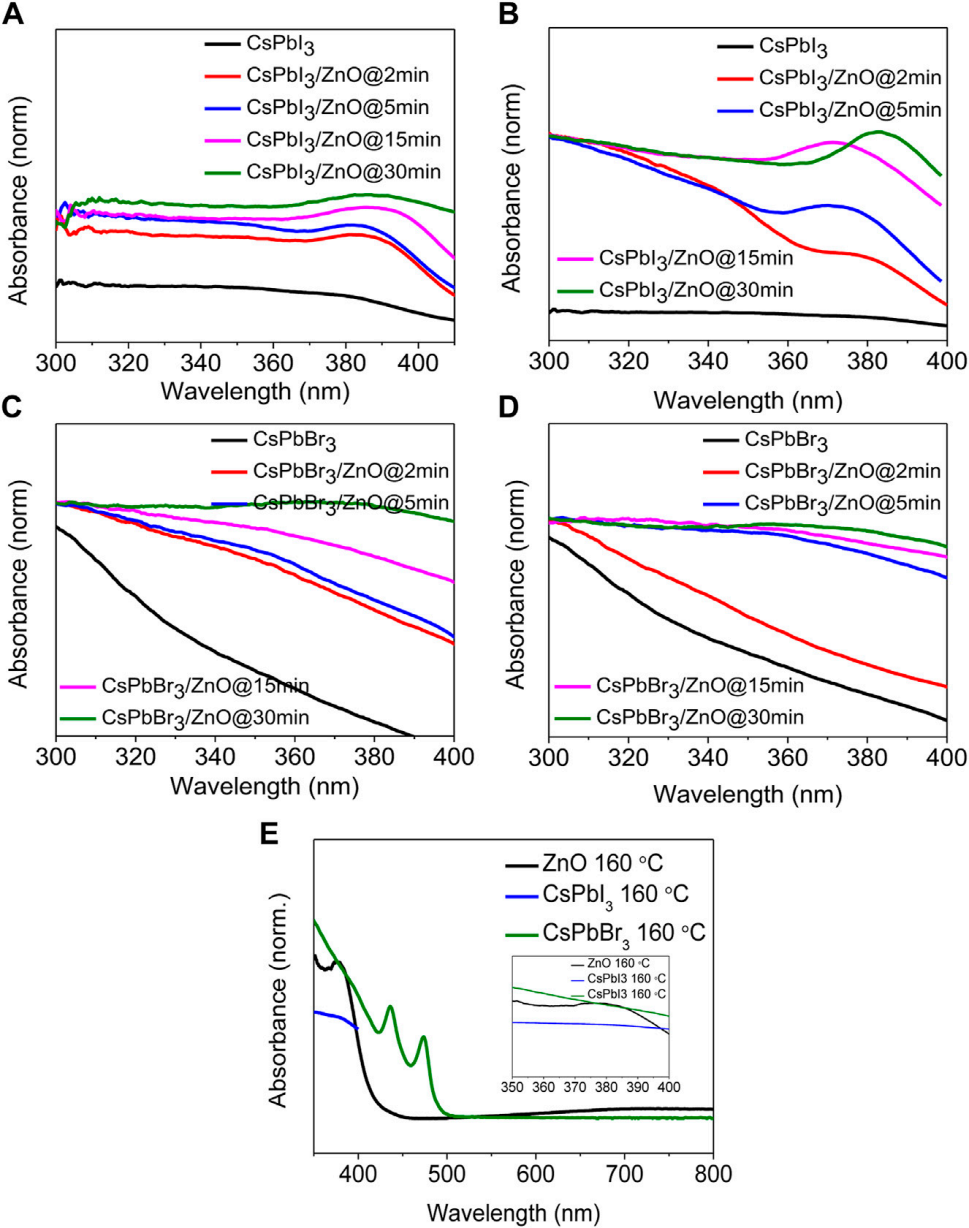
Absorbance spectra of inorganic CsPbI_3_ and CsPbI_3_/ZnO heterostructures at **(A)** 100°C and **(B)** 160 C. Absorbance spectra of inorganic CsPbBr_3_ and CsPbBr_3_/ZnO heterostructures at **(C)** 100 C and **(D)** 160 C. **(E)** Absorbance spectra for the controlled samples of CsPbI_3_ and ZnO. Inset shows the close view.

The UV-visible absorption spectra of two different inorganic lead halide perovskite (CsPbI_3_ and CsPbBr_3_) with ZnO as a photocatalyst for two temperatures, low (100°C) and high (160°C), with activity times ranging from 2 to 30 min, as shown in Figures 4A–D. Figures 4A,B shows that CsPbI_3_ possesses the highest photocatalytic activity, whereas Figures 4C,D represents that CsPbBr_3_ has no effect on ZnO even at elevated temperatures. It was observed that pristine CsPbBr_3_ has negligible photocatalytic activity due to obvious surface defects that introduce shallow transition levels and act as charge recombination sites, and also the material’s wider bandgap, which limits its use as a photocatalytic material. In comparison to CsPbBr_3_, CsPbI_3_ has a narrow bandgap of 1.73 eV and higher emission intensity, exhibiting the potential for excessive photo-generated ehp for photocatalysis (Tang et al., 2019; Zhang et al., 2019; Wang et al., 2021). ZnO’s good electron mobility, high absorption tendency, and cost-effectiveness make it an ideal photocatalyst. Coupling ZnO with halide perovskites enhances the generation of ehp by reducing the formation energy of the perovskites (Senapati et al., 2012; Jiang et al., 2019; Deng et al., 2020). Figures 4A,B show an absorbance peak from 370–385 nm due to zinc adsorption, confirming the presence of ZnO (Das et al., 2020; Karami et al., 2020), whereas, in Figures 4C,D show no change in the spectra, indicating that there is no zinc adsorption. Figures 5A,B depicts the obvious zinc adsorption in the CsPbI_3_/ZnO heterostructure by depicting the obvious absorbance peaks from 370–385 nm at both temperatures as compared to controlled samples of CsPbI_3_ and ZnO (Figure 5E), while Figures 5C,D represent no discernible change in the spectra. So, here we calculated the degradation efficiency with time in synthesized samples of CsPbI_3_/ZnO heterostructure at high temperature yielding a degradation efficiency of 52% by using Eq. 1 shown in Figure 4E.

The cycling stability of a photocatalyst is important for practical usage. Therefore, recycling experiments are carried out for CsPbI_3_/ZnO. The results are shown in Figure 5C suggested that the catalyst’s performance is negligible after four continuous cycles of photocatalyst reutilization. This study confirms that CsPbI_3_/ZnO is highly stable to heavy metal phtodegradation. The post-xps analysis indicted that the peaks for all the core elements are greatly reduced with decreasing intensity, confirming the photodegradation of heavy metals (d).

### 3.3 Cyclic voltammograms and AC impedance analysis of the CsPbI_3_/ZnO heterostructure

**FIGURE 6:**
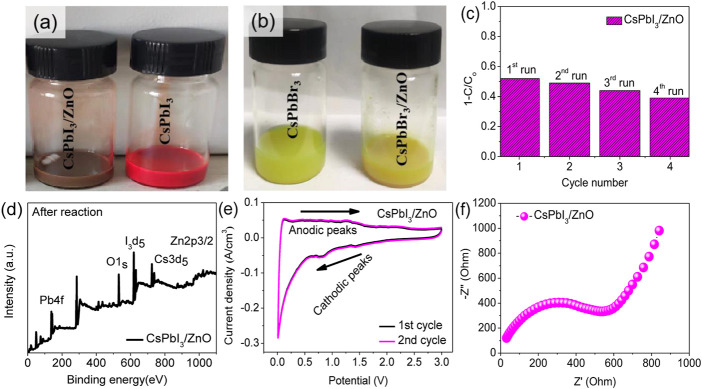
Adsorption of **(A)** CsPbI_3_/ZnO and CsPbI_3_, and **(B)** CsPbBr_3_/ZnO and CsPbBr_3_ heterostructure. **(C)** Cyclic stability of CsPbI_3_/ZnO under UV light. **(D)** Post-XPS analysis of CsPbI_3_/ZnO **(E)** Cyclic voltammograms of CsPbI_3_/ZnO at a scanning rate of 0.1 mV s^−1^ and **(F)** Nyquist plot at room temperature for CsPbI_3_/ZnO heterostructure.

The electrochemical performance of CsPbI_3_/ZnO was assessed to confirm its potential as a photocatalyst. In Figure 6E, the oxidative and reductive properties of the CsPbI_3_/ZnO heterostructure were investigated using the characteristic cyclic voltammogram test at a scanning rate of 0.1 mV s^−1^. The CV curve for the first cycle presented characteristic discharge peaks at 0.82 and 1.41 V, which are the reduction peaks that confirm the successful reaction of CsPbI_3_/ZnO heterostructure. Moreover, cathodic peaks confirm electrolyte decomposition and the formation of an SEI passivation layer on the surface of anode material during discharge. Two oxidative peaks were observed in the anodic sweep at 1.25 V, corresponding to a gradual Zn^+^ withdrawal process in CsPbI_3_/ZnO. The CV test confirms the oxidation and reduction potentials of CsPbI_3_/ZnO heterostructure. The electrochemical impedance spectra with a wide-range of frequency as shown in Figure 6F. The Nyquist curve consists of a semicircle and an oblique line from high to medium-frequency and high to low-frequency regions, respectively. The semicircle in the high-frequency region defined the extent of resistance to electron transfer, confirming the negligible ion transformation and bulk polarization, while the straight line represents the high electrical conductivity of the CsPbI_3_/ZnO catalyst. The result showed that the CsPbI_3_/ZnO catalyst had high electrical conductivity and was conductive to zinc ion diffusion.

### 3.4 Photocatalytic mechanism

**FIGURE 7:**
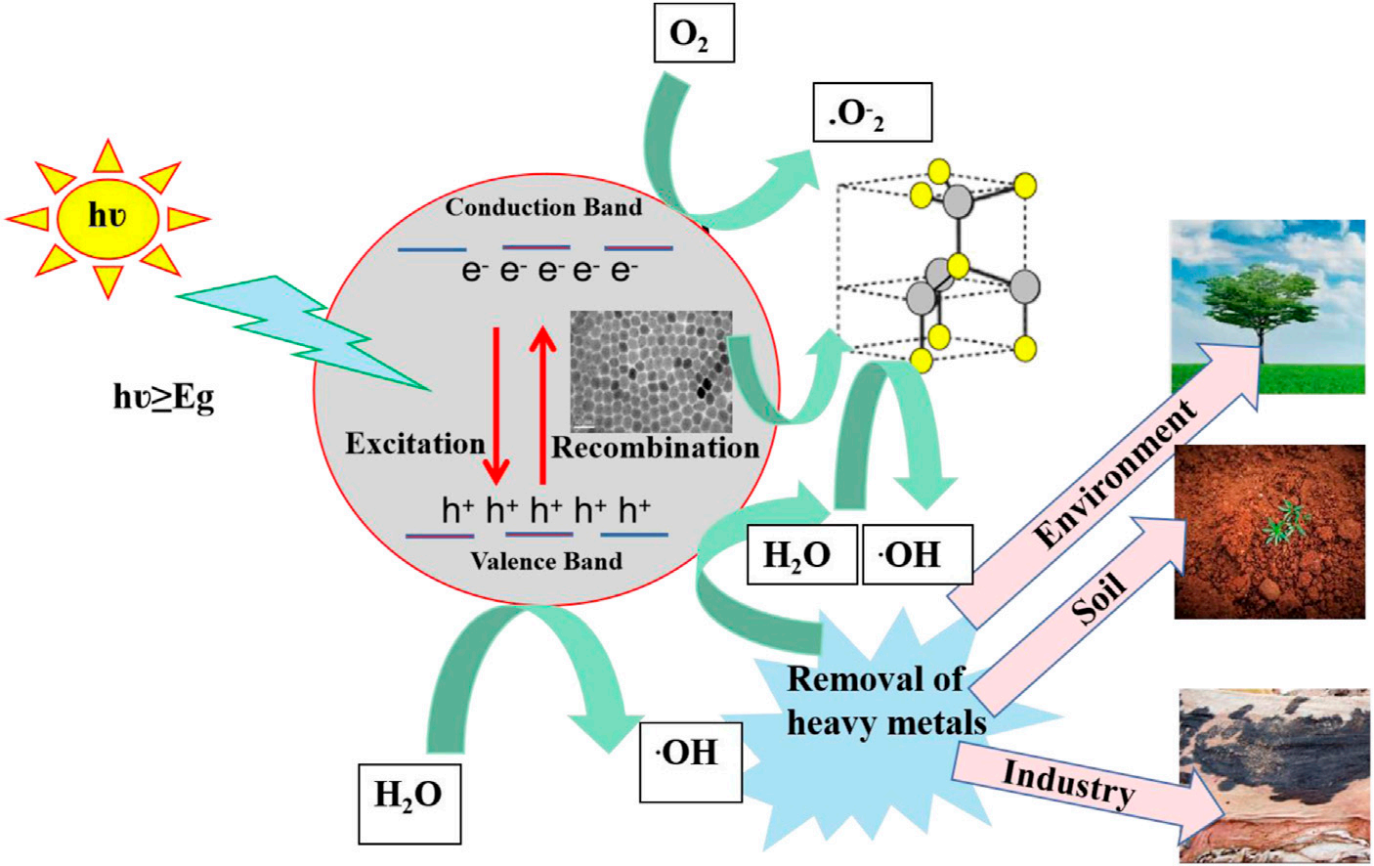
Schematic representation of the photocatalytic mechanism of CsPbX_3_/ZnO heterostructure.

Figure 7 depicts the photocatalytic mechanism of CsPbX_3_/ZnO heterostructure. Generally, the photocatalytic process initiates with the generation of electron hole pairs (ehp) in the presence of light with an energy greater than or equal to the bandgap (Wang et al., 2022). The electrons from the valence band (VB) are excited towards the conduction band (CB) of perovskites, thereby generating photo-active species including e^−^ and h^+^. Such photogenerated e^−^ would either combine with the h^+^ or be arbitrarily shifted to the surface of the photocatalysts, further trapped by O_2_ to generate O_2_^**.-**^.for the further generation of **.** OH (Wei et al., 2021). The formation of heterostructure has been regarded as an effective strategy for the retardation of the e/h recombination by keeping the utmost redox potential of a photocatalyst (Shen et al., 2022). Particularly, in this case of perovskite heterostructure, the generation of ehp occurs on the surface of CsPbX_3_ when an electron (eˉ) in the CsPbX_3_/ZnO shifts (excites) from the valence band (VB, HOMO) to the conduction band (CB, LUMO) and leaves holes (h^+^) in the HOMO region, known as the generation of ehp. In the CsPbX_3_ perovskite, these as-produced ehp react with H_2_O and O_2_, generating excessive hydroxyl radicals (^.^OH) and oxygen molecules (O_2_^−^) (Zhang et al., 2019). After the introduction of ZnO, holes can be shifted from the halide perovskite to the surface of ZnO, generating (^**.**^OH). The overall mechanism can be summarized as follows:

$$CsPbX_{3}+h\;\to e^{-}+h^{+},$$ (2)

$$O_{2}+e^{-}\;\to .O_{2}^{-},$$ (3)

$$H_{2}O+h^{+}\;\to .OH+H^{+},$$ (4)

$$ZnO+H^{+}\;\to {Zn}^{+}+\cdot OH$$ (5)

Thus, the produced radicals help in the reduction of heavy metals into environment-friendly end products like CO_2_ and H_2_O (Das et al., 2020; Khan and Pathak, 2020). Hence, these synthesized CsPbX_3_/ZnO heterostructures are quite useful in removing heavy metals and toxic products from the environment, soil, and different industry.

## 4 Conclusion

In conclusion, we have successfully synthesized the CsPbX_3_/ZnO heterostructure with zinc oxide as a photocatalyst using a standard hot injection method. The as-synthesized material was characterized by using different structural characterizations to confirm the successful formation and its morphological structure. The CsPbI_3_/ZnO heterostructure exhibits a degradation efficiency of 52%, which is higher than the degradation efficiency of CsPbI_3_ with various other dyes. The CV and EIS analysis show high oxidation and reduction characteristics, as well as superior resistance to electron transfer, confirming the electrical conductivity. Thus, this synthesized heterostructure has the potential to be used for heavy metal degradation as well as a water cleaning catalyst.

## Data Availability

The original contributions presented in the study are included in the article/Supplementary Material, further inquiries can be directed to the corresponding author.
